# The difference in effective light penetration may explain the superiority in photosynthetic efficiency of attached cultivation over the conventional open pond for microalgae

**DOI:** 10.1186/s13068-015-0240-0

**Published:** 2015-03-26

**Authors:** Junfeng Wang, Jinli Liu, Tianzhong Liu

**Affiliations:** Key Laboratory of Biofuels, Qingdao Institute of Bioenergy and Bioprocess Technology, Chinese Academy of Sciences, Qingdao, Shandong 266101 People’s Republic of China; Low-Carbon Energy Conversion Science and Engineering Center, Shanghai Advanced Research Institute, Chinese Academy of Sciences, Shanghai, 201203 People’s Republic of China

**Keywords:** Microalgae, Photosynthetic efficiency, Open pond, Attached cultivation, Effective light penetration

## Abstract

**Background:**

The ‘attached cultivation’ technique for microalgae production, combining the immobilized biofilm technology with proper light dilution strategies, has shown improved biomass production and photosynthetic efficiency over conventional open-pond suspended cultures. However, how light is transferred and distributed inside the biofilm has not been clearly defined yet.

**Results:**

In this research, the growth, photosynthetic oxygen evolution, and specific growth rate for microalgal cells in both open-pond and attached cultivation were studied to determine the effective light penetration at different phases of the cultivation. As a result, the light conditions inside the culture broth as well as the biofilm were revealed for the first time. Results showed that outdoor, in a conventional 20-cm deep open pond, all of the algal cells were fully illuminated in the first 3 days of cultivation. As the biomass concentration increased from day 4 to day 10, the light could only effectively penetrate 45.5% of the open-pond depth, and then effective light penetration gradually decreased to 31.1% at day 31, when the biomass density reached a maximum value of 0.45 g L^−1^ or 90 g m^−2^. In the attached cultivation system, under nitrogen-replete condition, almost 100% of the immobilized algal cells inside the biofilm were effectively illuminated from day 0 through day 10 when the biomass density increased from 8.8 g m^−2^ to 107.6 g m^−2^.

**Conclusion:**

Higher light penetration efficiency might be the reason why, using attached cultivation, observed values for photosynthetic efficiency were higher than those recorded in conventional open-pond suspended cultures.

## Background

Microalgae are a group of photosynthetic microorganisms represented by more than 40,000 species, many of which can produce high-value bioactive compounds [1]. In recent years, due to the price increase of fossil fuel as well as the concerns on environment deterioration, the global R&D investment on microalgae biofuel vastly increased. The reasons for this are that microalgae have high photosynthetic efficiency and high oil content and are believed to be the most promising feedstock for environment-friendly renewable liquid biofuel thanks to the fact that they do not compete with food crops for arable land and water supply [2-7]. However, until now, the success of microalgae cultures only happened in some small-scale tests and no success in biofuel production or CO_2_ mitigation has yet been achieved on a commercial scale anywhere in the world with microalgae cultivation [8,9]. The primary reason for this is that even when state-of-the-art techniques and designs are adopted on a large scale for open ponds or closed photobioreactors (PBRs), microalgal cell growth rate values currently cannot reach those obtained in highly controlled lab environments. The highest recorded biomass productivities for open ponds and PBRs are around 40 g dry mass m^−2^ day^−1^ and long-term averaged productivity is 10~20 g dry mass m^−2^ day^−1^ [3,10,11], which are far less than the theoretical maximum of 120~150 g m^−2^ day^−1^, or photosynthetic efficiency of 12.4% (based on total solar radiation spectrum) and 28% (based on visible light spectrum, 400~700 nm) [12,13].

**Figure 1:**
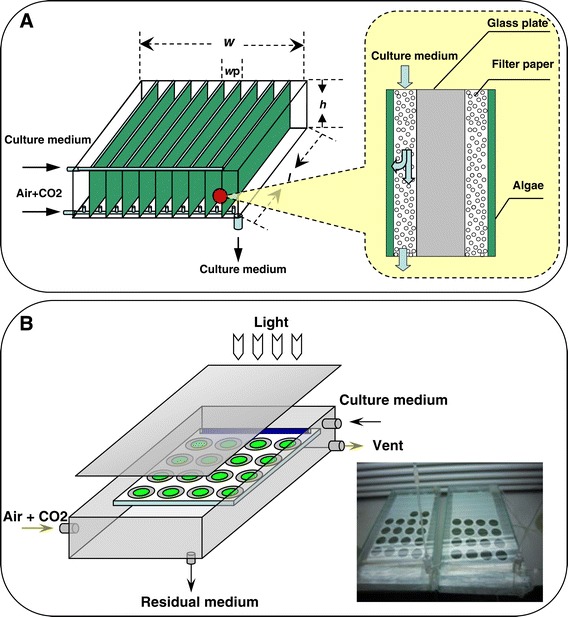
**The schematic diagrams of the photobioreactors for attached cultivation. (A)** The schematic diagram for the multiple-layer photobioreactor for attached cultivation (adapted from Liu *et al*. [22]). **(B)** The schematic diagram and the actual photograph for the single-layer photobioreactor for attached cultivation used in the research.

The biofilm cultivation of microalgae, in which the algal cells are generally immobilized and fixed onto/into supporting materials in high density and fed with nutrient solutions, is a different cultivation method than conventional aqueous suspended cultures. Open ponds and PBRs have existed for a long time now; however, they often exhibited relatively low biomass productivity [14-21]. Recently, our research group proposed an improved cultivation method based on a reactor where both the immobilized biofilm technology and the light dilution theory work together in a patented design called ‘attached cultivation’ [22]. The basic principles of this ‘attached cultivation’ technique as well as the related photobioreactors include the following: (i) a highly dense wet algal paste attached onto artificial supporting materials to form a thin layer of the algal biofilm and (ii) many layers of these biofilms arranged in an array fashion to dilute the high light so that the light intensity impinging the algal biofilm is much lower than the light that reaches the photobioreactor footprint area (Figure 1A). The biomass productivity potential of this ‘attached cultivation’ method was evaluated both under indoor and outdoor conditions. Results showed that with indoor light of 700 μmol m^−2^ s^−1^ and a light dilution rate of 10, the maximum biomass productivity for *Scenedesmus* was close to 120 g m^−2^ day^−1^, with photosynthetic efficiency of *ca.* 18% (based on visible light). Under outdoor conditions, the maximum biomass productivity reached *ca.* 80 g m^−2^ day^−1^ corresponding to a photosynthetic efficiency of 17.3% (based on visible light) and 8.3% (based on total solar irradiation), which were both seven times higher than the data reported for a conventional open pond under the same environment conditions [22]. Similar biomass productivity and photosynthetic efficiency were also achieved with *Botryococcus braunii* which grows slowly with aqueous suspended cultivation but exhibits a high biomass productivity of *ca.* 50 g m^−2^ day^−1^ with attached cultivation, corresponding to a photosynthetic efficiency of *ca.* 15% (based on visible light) [23].

As shown in these results, the ‘attached cultivation’ technique greatly improves the biomass productivity as well as photosynthetic efficiency of microalgae. However, the reason(s) and mechanism(s) for the superiorities of this method over suspended cultivation have not been fully understood yet. Maybe the secret of its superior performances over traditional suspended cultures lies in the differences in delivery of light and nutrients and in CO_2_ transfer between the two systems [24,25]. In this research, we especially focused on how the illumination worked, because light is always considered as the most important environmental factor that determines the behaviors of the microalgal cells [12,26]. A basic but important aspect to consider is how deep light penetrates during the microalgal cultivation. For open-pond suspended cultures, it is obvious that with the increases of biomass density, the light penetration depth decreases [12] and results in less and less percentage of algal cells being illuminated with light intensity which is higher than the compensation point (effectively illuminated) where the biomass accumulation rate due to photosynthesis equilibrates with the biomass loss rate due to respiration at a specific biomass density [11]. However, until to now, the dynamics of the ‘light’ *vs*. ‘dark’ for the open pond has not been quantitatively assessed. The information on illumination properties of the immobilized biofilm cultivation is scarce. How does the light transfer and penetrate inside the immobilized microalgal biofilm? Does light transfer and penetrate differently in the microalgal biofilm and in a suspended culture? How is the difference(s) related with the performances in photosynthetic efficiency? To answer these questions, the growth, photosynthetic oxygen evolution property, and specific growth rate for microalgal cells in open-pond and ‘attached cultivation’ systems were studied to determine the dynamics of effective light penetration (*d*_E_, depth from the surface where the light intensity is equal to the light compensation point) during cultivation. The light condition inside the outdoor open pond as well as the biofilm was revealed, and the effect of light distribution on the photosynthetic efficiency was discussed.

## Results and discussion

### The light distribution inside the suspended cultivation system

**Figure 2:**
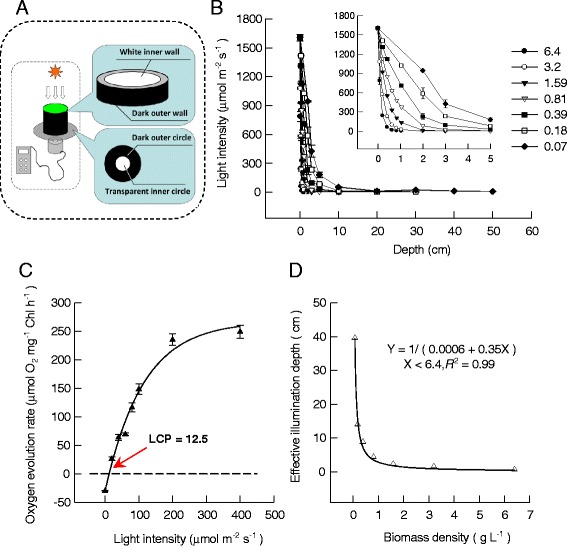
**The light attenuation inside the culture broth of suspended cultivation of** ***Scenedesmus dimorphus*** **. (A)** The schematic diagrams of light intensity measurements under different depths of the culture broth. **(B)** The dependence of light attenuation on culture density as well as depth. Seven different culture densities were tested, *viz.* 6.4 (black circle), 3.2 (white circle), 1.59 (black down-pointing triangle), 0.81 (white down-pointing triangle), 0.39 (black square), 0.18 (white square), and 0.07 (black diamond). The measurements were carried out outdoors with natural light. Data were mean ± standard deviation of three measurements. **(C)** The relationship of oxygen evolution rate *versus* light intensity (black triangle). Data were mean ± standard deviation of three measurements. The light compensation point (LCP) was indicated by arrows. **(D)** The effective illumination depth of aqueous suspended *S. dimorphus* culture broth at different biomass densities (white triangle).

The light intensity inside the open pond declined with the increase of broth depth as well as biomass concentration (Figure 2B). Similar results had been reported by Tredici [12]. According to the oxygen evolution properties, light intensity of 12.5 ± 3.9 μmol m^−2^ s^−1^ was considered as the light compensation point (LCP) for *Scenedesmus dimorphus*; under these circumstances, the oxygen consumption due to respiration already surpassed the oxygen evolution due to photosynthesis (Figure 2C). It should be noticed that the photosynthesis-light intensity (PI) curve of Figure 2C was measured on healthy vegetative algal cells that had been cultivated in the outdoor open pond for 5 days. According to our pilot experiment, from day 0 to day 30, there were non-significant differences among LCPs of algal cells from the outdoor open pond supplied with a non-nitrogen-deficient nutrient solution. The *d*_E_ for any given biomass density can be estimated from the results of light attenuation and LCP (Figure 2D). Accordingly, the *d*_E_ was only 0.57 cm at a biomass density of 5.0 g L^−1^, which means that only 40.4% of cells were effectively illuminated for a glass column having a diameter of 5 cm. In these conditions, the maximum biomass density would be 5.0 g L^−1^ after 10 days with continuous illumination of 100 μmol m^−2^ s^−1^ under indoor conditions. This *d*_E_ value might also be affected by light conditions, for example, the *d*_E_ might decrease in cloudy weather. To avoid this uncertainty, in this experiment, *d*_E_ measurement was performed under outdoor conditions with natural sunlight intensity of 1,500~1,600 μmol m^−2^ s^−1^ so that this equation could be directly applied to outdoor cultivation.

**Figure 3:**
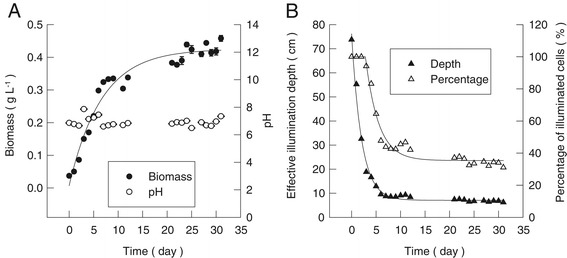
**The biomass increase, pH, and effective illumination depth of conventional aqueous suspended open ponds outdoors. (A)** The biomass density (black circle) and pH (white circle) changes of the open pond outdoors. Data for biomass were mean ± standard deviation of six measurements for two independent open ponds (three measurements for each). Data for pH were mean ± standard deviation of two measurements for two independent open ponds (one measurement for each). **(B)** The effective illumination depth (black triangle) and percentage of effective illuminated algal cells (white triangle) at different days of cultivation in outdoor open ponds.

In this study, we proved that for a conventional 20-cm deep open pond, the maximum biomass density would reach 0.45 g L^−1^ in 10 days and remain stable in the following days (Figure 3A). This maximum biomass density of 0.45 g L^−1^ in the open pond, corresponding to 90 g m^−2^, was similar with the results of Chisti [11] where the biomass density for open pond could not exceed 0.5 g L^−1^. The pH during the cultivation slightly fluctuated in the range of 6.7 ~ 7.5, indicating that the carbon source was not limited during the experiment (Figure 3A). According to the equation in Figure 2D, thanks to the low biomass concentration, during the first 3 days of cultivation, light could easily penetrate the suspended culture delivering optimal light intensity to each algal cell. After 31 days, only 31.1% of the algal cells were effectively illuminated, with the effective *d*_E_ being only 6.2 cm. In this research, the fastest biomass accumulation for the open pond was recorded from day 3 to day 7, corresponding to an increase of the biomass concentration from 0.1 to 0.3 g L^−1^ or areal biomass density from 20 to 60 g m^−2^. During the same time window, biomass productivity reached *ca.* 10 g m^−2^ day^−1^, and the effective light penetration continuously decreased from 100% to 45.5% (Figure 3B). The biomass productivity did not decrease with the reduced effective illumination depth during these times but remained at 10 g m^−2^ day^−1^. In microalgal suspended cultures, light distribution is not uniform at all. Light intensity decreases exponentially as we move farther away from the illuminated surface; for this reason, a thin layer on the water surface usually receives oversaturating light intensity while the bottom of the pond, as the algae cells reproduce and the biomass concentration increases, lies in total darkness. The algal cells traveling through the dark portion of the reactor consume biomass by dark respiration, if they spend a long enough time in this kind of environment. An appropriate depth of the water layer in an open pond is required to avoid biomass loss by cellular respiration. The optimal depth of any suspended culture of algae cells should be equal to the maximum depth at which a sufficient amount of solar light can penetrate. The aforementioned values for how ‘light’ and ‘dark’ regions expand and contract as the culture grows thicker are very likely to change depending on the different geographic locations considered other than Qingdao, China, where this experiment was conducted and the particular algal strain considered or the reactor design adopted. Identifying the upper limit of biomass density and the maximum depth at which algae cells are still effectively illuminated depending on the specific design of the reactor (different thicknesses of the water layer) has been an interesting task for our research team. Obtaining this information will definitely help to better understand how light travels through the water layer of an open pond, and it will also be useful knowledge for enhancing biomass productivity.

### The light distribution inside the biofilm of the attached cultivation system

**Figure 4:**
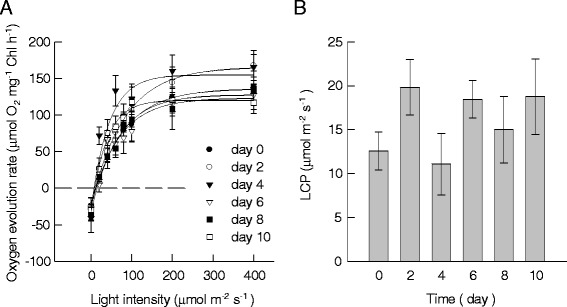
**The oxygen evolution characters of** ***Scenedesmus dimorphus*** **under attached cultivation for different days of cultivation. (A)** The oxygen evolution rate versus light intensity (light curve) of *S. dimorphus* under attached cultivation for 0 (black circle), 2 (white circle), 4 (black down-pointing triangle), 6 (white down-pointing triangle), 8 (black square), and 10 (white square) days of cultivation. Data were mean ± standard deviation of six measurements for three independent experiments (two measurements for each). **(B)** The light compensation point of *S. dimorphus* under attached cultivation for different days of cultivation. The ANOVA (SPSS, Chicago, IL, USA) result indicated that there were no differences for different days.

One of the methods to determine the *d*_E_ of the attached microalgal biofilm is to directly measure the light intensity from underneath the biofilm. If the light intensity was equal to or higher than the LCP, then the corresponding depth could be effectively illuminated. However, we must also consider that the photosynthetically active wavelengths (red light) are more prone to be absorbed by chlorophyll than other less desired wavelengths (green light). The method we just described to measure light intensity might overestimate the *d*_E_ if only the quantum flux intensity is measured because the undesired wavelengths travel farther away through layers of cells than the desired ones. To avoid the aforementioned problem in this research, we used a different method to estimate the *d*_E_ by monitoring the specific growth rate of a thin layer of microalgal cells, which was called ‘marker’ layer, inserted just beneath the re-constructed biomass layer. An optimum ‘marker’ layer should have two main characteristics, *viz*. 1) thin enough to avoid the formation of light gradient inside the layer and 2) sensitive enough to the changes in light intensity. Algal cells from this thin layer should experience the same conditions as the upper layer. However, in pilot experiments, we found that such thin marker layers failed to meet requirement #2. In other words, the biomass changes for this kind of thin layers were too slight to be detected by regular gravimetric method, especially under lower light conditions. As an alternative, the algal cells used to inoculate the attached cultivation were chosen as the ‘marker’ layer because they were photosynthetically active even at low biomass densities. However, the risk of over- or underestimating the *d*_E_ with this method was still present if the LCP of the inoculum algal cells was lower or higher than the LCPs of daughter cells that only experienced attached cultivation conditions. The LCP for the newly formed microalgal cells was firstly measured at different days. Typical light curves for oxygen evolution rate were obtained for all of these detached and re-suspended samples (Figure 4A). The LCPs of the daughter cells from day 0 to day 10 were in the range of 12.6 ± 2.2 ~ 19.8 ± 3.2 μmol m^−2^ s^−1^, without significant differences (*P* > 0.05) (Figure 4B). These results indicated that it is safe to determine the *d*_E_ of the attached algal biofilm with this double-layer method under nutrient-replete condition without worrying about over- or underestimation. We also tested the LCP changes for the attached algal biofilm under nitrogen-depleted conditions and found that it increased dramatically to *ca.* 60 μmol m^−2^ s^−1^ at day 2. Because of this, a ‘marker’ layer consisting of the cells used to inoculate the attached cultivation surface cannot be used to estimate the *d*_E_ of the nitrogen-starved attached cultivation.

**Figure 5:**
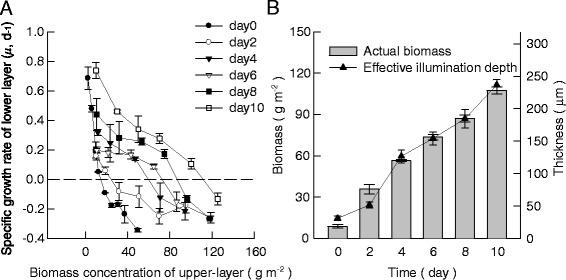
**The estimation of effective illumination depth for the attached cultivation of** ***S*** **.** ***dimorphus*** **with twin-layer method. (A)** The specific growth rate of lower layer *versus* the upper layer biomass density. Data were mean ± standard deviation of nine measurements for three independent experiments (three measurements for each). The upper layers were consisted with biomass grown with attached cultivated technique for 0 (black circle), 2 (white circle), 4 (black down-pointing triangle), 6 (white down-pointing triangle), 8 (black square), and 10 (white square) days. **(B)** The actual biomass density for the attached cultivation from day 0 to day 10 (closed bar), and the effective illumination depth for the attached cultivation from day 0 to day 10 (black triangle).

The results of the double-layer experiments for attached cultivation of *S. dimorphus* under nitrogen-replete condition are shown in Figure 5. For the biofilm layer that has been attached cultivated for several days, the specific growth rate of the ‘marker’ layer (*μ*) decreased as the biomass of the upper layer increased, mainly because the light traveling through the upper layer became weaker and weaker (Figure 5A). Meanwhile, the rate of this decrease for *μ* gradually slowed down as the days went by, resulting in the increase of the thicknesses for the effectively illuminated upper layers (Figure 5A). According to the results in Figure 5A, the *d*_E_ for attached cultivation under nitrogen-replete condition increased from 33.9 ± 6.5 μm for day 0 to 237.3 ± 11.8 μm for day 10, corresponding respectively to biomass densities of 14.6 ± 1.6 g m^−2^ and 111.5 ± 4.1 g m^−2^, whereas the actual biomass density increased from 8.8 ± 1.3 g m^−2^ to 107.6 ± 2.6 g m^−2^ in 10 days, corresponding to thicknesses of 21.7 ± 5.9 μm and 229.1 ± 8.6 μm (Figure 5B), respectively. The ratios of *d*_E_*vs*. actual biomass were almost equal to 1 throughout the experiments except for day 0 and day 2 which were 1.66 and 0.68, respectively. The fact that *d*_E_ was similar to the actual biomass density indicated that nearly 100% of the algal cells inside the immobilized biofilm were effectively illuminated under attached cultivation.

**Figure 6:**
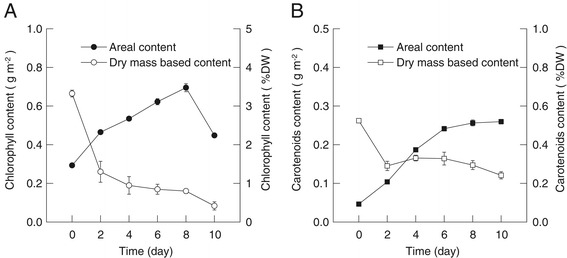
**The chlorophyll and carotenoid contents for the attached cultivation of** ***S*** **.** ***dimorphus*** **. (A)** The areal (black circle) and dry mass-based (white circle) chlorophyll contents for attached cultivation of *S. dimorphus* for different days of cultivation. Data were mean ± standard deviation of six measurements for three independent experiments (two measurements for each). **(B)** The areal (black square) and dry mass-based (white square) carotenoid contents for attached cultivation of *S. dimorphus* for different days. Data were mean ± standard deviation of six measurements for three independent experiments (two measurements for each).

**Figure 7:**
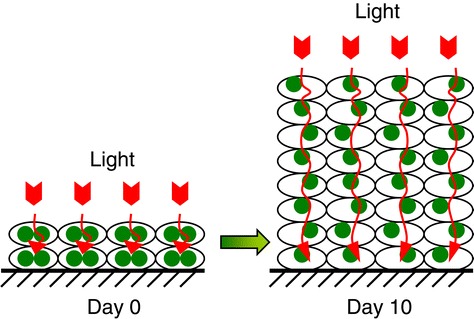
**The schematic model for the chlorophyll and light distribution inside the attached cultivation biofilm of** ***S*** **.** ***dimorphus*** **.** The small oval represents the algal cells inside the biofilm. The green dots indicate the chlorophyll content of algal cells. The suggested light penetration path inside the biofilm is indicated by red arrows.

Chlorophyll is the main light-harvesting molecule for photosynthetic organism, but some of the carotenoid molecules can also capture light and pass the excited energy to chlorophyll [27]. In this research, the content of chlorophyll as well as carotenoid was measured and values have been expressed as both a function of the dry biomass and the cultivated surface area. From day 0 to day 10, the dry mass-based chlorophyll content decreased from 3.3% to 0.4% and the carotenoid content decreased from 0.5% to 0.2%. The areal chlorophyll content increased from 0.29 to 0.70 g m^−2^ during the first 8 days of attached cultivation and then decreased to 0.45 g m^−2^ at day 10. The areal carotenoid content increased from 0.05 to 0.26 g m^−2^ from day 0 to day 8 and then remained constant. In general, during the attached cultivation, the increase of the *d*_E_ was accompanied by the decrease in biomass-based chlorophyll and carotenoid contents as well as the increase in areal chlorophyll and carotenoid contents (Figure 6). Figure 7 suggests how the light transfer and distribution inside the immobilized microalgal biofilm are thought to be happening. In the earlier phase of attached cultivation, the pigment content in every single cell is relatively high, so that the light intensity decreased sharply when passing through each algal layer and can penetrate only a small depth of the algal biofilm. With the increase of the cultivation days, the algal layers as well as the pigment content along the light path increased but the pigment content in each single cell decreased, and only a moderate decrease in light intensity happens when passing through each algal layer. In the end, light can penetrate a bigger depth of the algal biofilm (Figure 7).

### Comparison of the illumination aspects of the suspended and attached cultivation

The obvious difference between the two cultivation systems was that full illumination only happened for a short period of time (day 0 to day 3) in the open-pond system, when biomass concentration was very dilute (<20 g m^−2^), whereas in the attached cultivation system, the full illumination happened during a prolonged interval of 10 days as well as a wider range of biomass density (up to 100 g m^−2^ for a single layer). This high percentage of photosynthetic active biomass might explain the high biomass productivity and photosynthetic efficiency of attached cultivation under high light conditions. For example, in our previous research with a multiple-plate attached cultivation bioreactor with a light dilution rate of 10 (refer to Figure 1A), the land areal biomass density increased from 163.7 to 484.5 g m^−2^ in 5 days [22] under outdoor high light conditions (1,500 ~ 2,000 μmol m^−2^ s^−1^). Since the averaged light intensity impinging the cultivated surface was only about 100 ~ 150 μmol m^−2^ s^−1^, we can assume that nearly 100% of the biomass in this case was effectively illuminated. Similar results were also achieved under indoor conditions. The high photosynthetic efficiency of *ca.* 18% (based on visible light) was reached for *Scenedesmus* indoors with a light intensity of 700 μmol m^−2^ s^−1^ in the land areal biomass density range of 192.5 to 828.1 g m^−2^ [22]. Photosynthetic efficiency of *ca.* 15% (based on visible light) was also reached for *Botryococcus braunii* indoors with a light intensity of 500 μmol m^−2^ s^−1^ in the standing areal biomass density range of *ca.* 70 to *ca.* 580 g m^−2^ [23]. It is hard to imagine an open pond working with such high percentage of photosynthetic active cells and consequently such a condensed biomass concentration range. In general, for the attached cultivation, the high light intensity is diluted and absorbed by a cultivated area hosting an extremely dense biomass, where there is virtually no space left between adjacent cells, which is totally photosynthetically active, whereas in conventional open ponds, the high light intensity is only absorbed by a much smaller amount of algal cells so that the average light energy received by each photosynthetically active cell is higher in the open pond and a large portion of light is dissipated in a non-photosynthetic pathway before reaching other cells.

## Conclusions

In a conventional 20-cm deep open pond, all of the algal cells were fully illuminated for the first 3 days of outdoor cultivation. With the increase of the biomass concentration, the effective illuminated portion of the water layer decreased steeply to 45.5% from day 4 to day 10 and then gradually decreased to 31.1% at day 31, when the biomass density reached 0.45 g L^−1^ or 90 g m^−2^. In the attached cultivation system under nitrogen-replete condition, almost 100% of the immobilized algal cells inside the biofilm were effectively illuminated from day 0 through day 10, with biomass density increasing from 8.8 to 107.6 g m^−2^. Attached cultivation facilitates super high biomass density (up to *ca.* 800 g m^−2^) and super high efficient illumination (100%) when the reactor consists of multiple panels hosting the biofilm surface which might be the key to the high biomass productivity as well as high photosynthetic efficiency.

## Methods

### Microalgae strain and medium

The freshwater microalgal species *S. dimorphus* was locally screened in Qingdao, China. The algal cells were maintained in BG11 medium [28], each liter of which contains 1.5 g NaNO_3_, 0.075 g MgSO_4_·7H_2_O, 0.036 g CaCl_2_·2H_2_O, 0.04 g KH_2_PO_4_·H_2_O, 0.02 g Na_2_CO_3_, 6.0 × 10^−3^ g citric acid, 1.0 × 10^−3^ g Na_2_EDTA, 6.0 × 10^−3^ g ferric ammonium citrate, 2.22 × 10^−4^ g ZnSO_4_·7H_2_O, 6.9 × 10^−5^ g CuSO_4_·5H_2_O, 1.81 × 10^−3^ g MnCl_2_·4H_2_O, 3.9 × 10^−4^ g Na_2_MoO_4_·2H_2_O, 4.94 × 10^−5^ g Co(NO_3_)_2_·6H_2_O and 2.86 × 10^−3^ g H_3_BO_3_.

### Inoculum preparation

The algal inoculum was prepared in glass columns (diameter = 0.05 m, working volume = 0.7 L) under indoor conditions of continuous illumination (100 μmol m^−2^ s^−1^; cold fluorescent lamp, white light; FSL T8 36W, Foshan Electrical Lighting Co., Ltd., Foshan, China) and continuous aeration (1.0 vvm; 98% air + 2% CO_2_, *v*/*v*). The temperature of the culture broth was 25°C ± 1°C. The algal biomass at late exponential phase (5~7 days after inoculation, 2~3 g L^−1^) was used to inoculate the experimental systems.

### Experimental design

**Figure 8:**
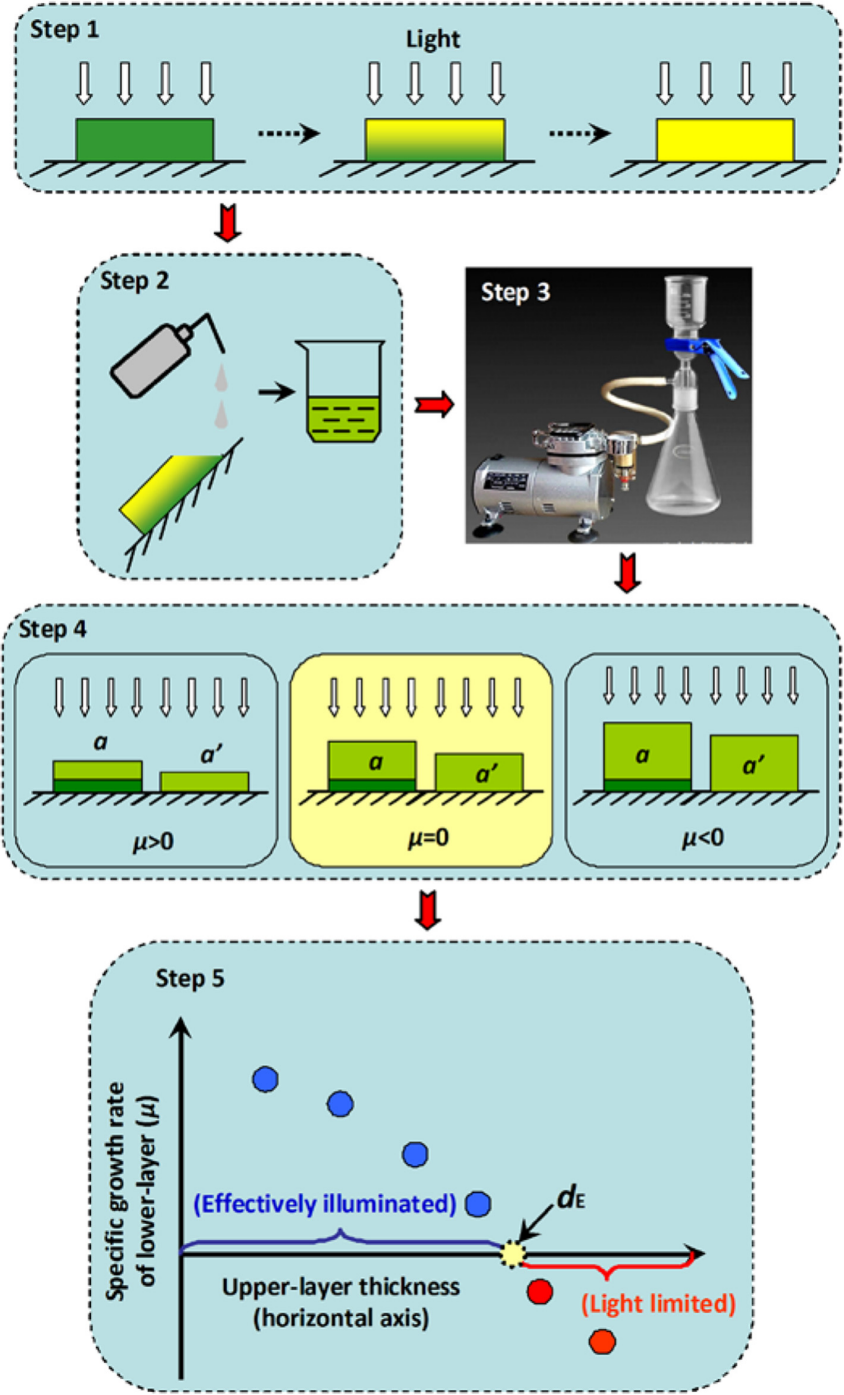
**The schematic flow chart of the experiment design to determine the effective illumination depth for the attached cultivation.**

The *d*_E_ for the algal biofilm of the attached cultivation and conventional aqueous suspended open pond were determined under indoor and outdoor conditions, respectively. For the attached cultivation, the *d*_E_ was measured with a single-layer attached cultivation system (Figure 1B) following the flow chart of Figure 8. Firstly, the algal cells were attached cultivated for 10 days with BG-11 (step 1; refer to the ‘Cultivation systems’ section for details). Every other day, some of the algal disks were sampled and the attached cells on it were detached and washed three times with de-ionized water (step 2). A ‘marker’ layer was built by filtering some fresh inoculum onto new cellulose acetate/nitrate membranes at the biomass density of *s* (g m^−2^). Then, some aliquots of the re-suspended algal culture from step 2 were gently filtered onto the ‘marker’ layers to form ‘double-layer’ attached algal disks. The corresponding ‘single-layer’ attached algal ‘disks’ were also prepared at the same time as a control using identical aliquots of re-suspended algal culture but without the ‘marker’ layer (step 3). Both of the newly prepared ‘double-layer’ and ‘single-layer’ algal disks were cultivated for 24 h under the same environmental conditions using the PBR depicted in Figure 1B. The biomass increase for the ‘double-layer’ algal disk was denoted by *a* (g m^−2^) and the biomass increase for the corresponding ‘single-layer’ algal disk was *a’* (g m^−2^), so that the net biomass gain for the ‘marker’ layer was calculated as *δ* = *a* − *a’* (g m^−2^), and *μ* was calculated as:

1 $$ \mu = \ln \left(s+\delta \right)- \ln (s) $$

If *μ* > 0, it means that the ‘marker’ layer is receiving a light intensity higher than the LCP. In other words, the upper layer is not thick enough to fully attenuate the impinging light and the depth of the upper layer (*d*_A_) was only a fraction of the *d*_E_, *d*_A_ < *d*_E_.

If *μ* < 0, it means that the light intensity reaching the ‘marker’ layer is lower than the LCP and the upper layer is too thick to allow the ‘marker’ layer to accumulate biomass, *d*_A_ > *d*_E_.

If *μ* = 0, the light intensity at the ‘marker’ layer is equal to the LCP and the biomass increase of the biofilm due to photosynthesis is equal to the biomass loss due to cellular respiration. The thickness of the upper layer could be considered as the maximum distance the light can effectively penetrate, *d*_A_ = *d*_E_ (Figure 8, step 4). In actual experiments, however, considering the difficulties in precisely manipulating the amount of the upper-layer biomass to make *d*_A_ = *d*_E_, the *d*_E_ was generally approximately estimated as the intercept of the *x*-axis (Figure 8, step 5).

The percentage of the effectively illuminated cells (*P*_light_, %) for attached cultivation was calculated as:

2 $$ {P}_{\mathrm{light}}={d}_{\mathrm{E}}\times 100/{d}_{\mathrm{G}} $$

where *d*_G_ represents the actual thickness of the attached algal ‘disk’ at the considered sampling day.

The *d*_E_ in the suspended cultivation system was determined with a conventional open-pond system and a set of homemade equipment with different light paths (Figure 2A; refer to the sections ‘Equipments for determining the effective illumination depth for suspended cultures’ and ‘Cultivation systems’ for detailed specifications and operations). Firstly, the relationships of biomass concentrations *vs.* penetrated light intensities were measured (Figure 2B). Secondly, according to the LCP determined by oxygen evolution rates at different light intensities (Figure 2C; refer to the ‘Determination of LCP’ section for details), the *d*_E_ were calculated for each tested biomass densities. An equation was proposed to calculate the *d*_E_ at any given biomass density in the range of 0.07 ~ 6.4 g L^−1^ (Figure 2D). Finally, the changes in biomass density of *S. dimorphus* in open ponds were studied outdoors (Figure 3A), and the *d*_E_ was estimated according to the proposed equation. The percentage of the effectively illuminated cells (*P*_light_, %; Figure 3B) was calculated as:

3 $$ {P}_{\mathrm{light}}={d}_{\mathrm{E}}\times 100/{d}_{\mathrm{P}} $$

where the *d*_P_ represents the depth of the open pond, 0.2 m in this research.

### Equipments for determining the effective illumination depth for suspended cultures

A series of white polyvinyl chloride (PVC) tubes (inner diameter = 0.03 m) with different lengths (2, 3, 5, 10, 20, 30, 40, and 50 cm; three tubes for each length) were prepared, and one of the open ends of these PVC tubes was covered with a piece of highly transparent polystyrene plate (thickness = 0.8 mm; >98% light transmittance). The outer wall of the PVC tubes and the outer circles of the polystyrene sheets were painted black to prevent the unwanted penetration of outside irradiance, whereas the inner wall and the inner circles (diameter = 1 cm) were not painted but thoroughly cleaned with water (Figure 2A). Algal suspensions with different biomass densities, that is, 0.07, 0.18, 0.39, 0.81, 1.59, 3.20 and 6.4 g L^−1^, were prepared in beakers and then used to fill the PVC tubes, so that the heights of the algal broth, *viz*. light paths, were equal to the lengths of the PVC tubes. The PVC pipes were placed vertically in open air, under direct sunlight with an intensity of approximately 1,500 ~ 1,600 μmol m^−2^ s^−1^. A photosynthetic quantum sensor (Li-250A, Li-Cor, Lincoln, NE, USA) was placed underneath each tube and tightly attached to the polystyrene sheets to measure the penetrated photon flux density.

When low water layer thicknesses were to be tested, that is, 0.084 ~ 1.05 cm, the algal suspension was poured into a Petri dish made of highly transparent polystyrene (thickness = 0.8 mm; >98% light transmittance) and then the penetrated light intensity was measured. The thickness of the water layer in the Petri dish was calculated according to the volume of algal suspension poured into and the diameter of the Petri dish used. The external surface of the Petri dish was also painted with black paint with the exception of a small circular window (diameter = 1 cm) where the quantum sensor was to be attached.

### Cultivation systems

Two different cultivation systems were adopted in this research to evaluate the active proportions for photosynthesis. The first one was a mini open-pond system which measured 2 m in length, 1 m in width, and 0.2 m in culture depth. The culture broth was propelled by a paddle wheel at a flow speed of 0.3 m s^−1^. A gas stream of pure CO_2_ (>99%, *v*/*v*) was injected into the bottom of the pond through a ventilation stone at an aeration speed of 4 L min^−1^ (0.01 vvm) from 8 am to 4 pm to supply the carbon source for the algal cells and maintain a pH of 7.5 ± 0.5. The measured maximum light intensity at the water surface was *ca.* 2,000 μmol m^−2^ s^−1^ during the daytime. The temperature of the culture broth was 30°C~35°C from 12 pm to 2 pm in the afternoon on a sunny day and 10°C~25°C for the rest of the time. Two identical ponds were operated synchronously in outdoor conditions at Qingdao, China (35°35′ N, 119°30′ E) from 17 September to 17 October 2012, with the same inoculated biomass density of 0.04 g L^−1^ (8 g m^−2^).

The second adopted cultivation system was the single-layer attached biofilm cultivation system (Figure 1B) which has been introduced in detail in our previous research as a ‘type 1’ photobioreactor [22]. In brief, algal cells were evenly filtered onto a cellulose acetate/nitrate membrane (pore size 0.45 μm) to form algal disks with 10 ± 0.5 cm^2^ of footprint at an inoculated areal biomass density of 8 g m^−2^. These algal disks were then put onto a layer of regular filter paper and finally onto a 0.2 m × 0.4 m glass plate (3 mm thick). The glass plate as well as the filter paper and algal disks on it were sealed in a 0.5 m × 0.3 m × 0.05 m chamber made out of 5-mm-thick transparent glass plates. During the cultivation, the algal disks were continuously illuminated with 100 ± 10 μmol m^−2^ s^−1^ of white light from cold fluorescent lamps. A continuous air stream that contained 1.5% (*v*/*v*) of CO_2_ was injected into the chamber with a flow rate of 0.75 L min^−1^ (0.1 vvm). The BG11 medium was dripped onto the filter paper with a speed of 0.5 mL min^−1^ to keep the algal disks attached to the filter paper and provide nutrients for the growth of the algal cells. Only fresh medium was used, and any excess was immediately discarded. The temperature inside the glass chamber was kept at 25°C ± 2°C during the experiment.

### Analysis

#### Growth analysis

In this research, the growth of the algal culture was investigated by measuring the changes in dry biomass concentration (DW). Some aliquots of the culture broth (open pond) or detached biomass from the ‘algal disk’ (attached cultivation) were first washed with 50 mL of de-ionized water and then filtered onto a pre-weighted 0.45-μm GF/C filter membrane (Whatman, Little Chalfont, England; DW_0_). The membrane was oven dried at 105°C for 24 h and then weighted (DW_1_).

The DW for open pond (g L^−1^) was calculated as:

4 $$ \mathrm{D}\mathrm{W}=\left({\mathrm{DW}}_1-{\mathrm{DW}}_0\right)\times 1,000/\nu $$

where *v* represents the sample volume expressed in milliliters.

The DW for attached cultivation was calculated as:

5 $$ \mathrm{D}\mathrm{W}=\left({\mathrm{DW}}_1-{\mathrm{DW}}_0\right)/0.001 $$

where 0.001 represents the footprint area (m^2^) of attached algal cells.

#### Determination of LCP

The LCP was determined according to the photosynthetic oxygen evolution rate *vs*. light intensity curve (PI curve); this was measured with a Chlorolab-2 liquid-phase Clark-type oxygen electrode (Hansatech, Norfolk, England). The newly prepared inoculum for the algal disks (refer to the ‘Inoculum preparation’ section) was collected by centrifugation at 5,000 × *g* for 30 s (Allegra X-22R, Beckman Coulter, Brea, CA, USA). The obtained pellet was then re-suspended with BG-11 medium containing 100 mM NaHCO_3_ and adjusted to a proper biomass concentration so that the chlorophyll content was as close as possible to 10 μg per milliliter. One milliliter of the sample was added to the reaction cuvette and then bubbled with nitrogen gas for 30 s to expel the dissolved oxygen gas. Light intensities of 400, 200, 100, 80, 60, 40, 20 and 0 μmol m^−2^ s^−1^ were applied to drive the photosynthetic oxygen evolution. The measurement was repeated three times for each light intensity. The LCP was obtained as the intercept on the horizontal axis of the PI curve [29].

#### Determination of the thickness of the attached algal disk

Prior to the experiments, the thickness of the algal disks at different cultivation days was measured by means of a surface profiler (Veeco Dektak150, Veeco Instruments Inc., Plainview, NY, USA) and the corresponding areal biomass density was also measured (refer to the ‘Growth analysis’ section). Accordingly, the correlation of the thickness (*d*, expressed in μm) of the algal film with the areal biomass density (DW, in g m^−2^) was obtained as follows:

6 $$ d=2.1\times \mathrm{D}\mathrm{W}+3.2 $$

For later experiments, the thickness (μm) of the attached algal disks during cultivation was estimated by this equation. According to the pilot experiments, this equation could be applied in the biomass range of 0 ~ 120 g m^−2^ for *S. dimorphus* grown with the attached cultivation technique for up to 10 days under both nitrogen-replete or nitrogen-depleted conditions (*R*^2^ = 0.99).

#### Determination of the chlorophyll and carotenoid contents

The chlorophyll and carotenoid contents were determined according to the methods described by Wellburn [30]. The algal biomass was harvested and washed with plenty of de-ionized water and then mixed with methanol under 60°C for 12 h in darkness until the algal cells whitened completely. The optical densities of the extraction were measured with a spectrophotometer at 666, 653, and 470 nm (Cary 50, Varian Inc., Palo Alto, CA, USA). The chlorophyll a (Chl_a_), chlorophyll b (Chl_b_), and carotenoid (Car) concentrations in the extraction were calculated as follows (mg L^−1^):

7 $$ {\mathrm{Chl}}_{\mathrm{a}}=15.65\times {\mathrm{OD}}_{666}-7.34\times {\mathrm{OD}}_{653} $$

8 $$ {\mathrm{Chl}}_{\mathrm{b}}=27.05\times {\mathrm{OD}}_{653}-11.21\times {\mathrm{OD}}_{666} $$

9 $$ \mathrm{Car}=\left(1,000\times {\mathrm{OD}}_{470}-2.86\times {\mathrm{Chl}}_{\mathrm{a}}-129.2\times {\mathrm{Chl}}_{\mathrm{b}}\right)/221 $$

## Abbreviations

*a*: biomass increase for the ‘twin-layer’ algal disk
*a’*: biomass increase for the corresponding ‘single-layer’ algal disk
Car: concentration of carotenoid
Chl_a_: concentration of chlorophyll a
Chl_b_: concentration of chlorophyll b
*D*: thickness of the algal film
*d*_A_: the depth of the upper layer
*d*_E_: effective illumination depth
*d*_G_: actual thickness of the attached algal ‘disk’ at different sampling days
DW: dry biomass concentration
DW_0_: dry weight of the filter membrane
DW_1_: dry weight of the filter membrane after filtering the algal cells
LCP: light compensation point
PBR: photobioreactor
PI curve: curve of photosynthetic oxygen evolution rate *vs*. light intensity
*P*_light_: percentage of the effectively illuminated cells
PVC: polyvinyl chloride
*V*: volume of the sample for biomass measurement
*δ*: net biomass gain for the ‘seed’ layer
*μ*: specific growth rate
